# Effects of Coaching-Based Teleoccupational Guidance for Home-Based Stroke Survivors and Their Family Caregivers: A Pilot Randomised Controlled Trial

**DOI:** 10.3390/ijerph192316355

**Published:** 2022-12-06

**Authors:** Li Zhang, Yan-Ning Yan, Zeng-Xin Sun, Dong-Rui Yan, Yuan-Wu Chen, Keh-Chung Lin, Xin-Jing Ge, Xiao-Lu Qin

**Affiliations:** 1 Graduate School, Hebei Medical University, Shijiazhuang 050051, China; 2 Department of Rehabilitation Medicine, Hebei General Hospital, Shijiazhuang 050051, China; 3 School of Occupational Therapy, College of Medicine, National Taiwan University, Taipei 10617, Taiwan; 4 Division of Occupational Therapy, Department of Physical Medicine and Rehabilitation, National Taiwan University Hospital, Taipei 10055, Taiwan

**Keywords:** coaching, home-based stroke rehabilitation, occupational therapy, stroke, telerehabilitation

## Abstract

The aim of this pilot study was to investigate the feasibility and effectiveness of a 3-month coaching-based teleoccupational guidance (CTG) programme for home-based stroke survivors and their family caregivers. An assessor-blind pilot randomised controlled study was conducted. Twenty-five participant dyads (each dyad consisted of one home-based stroke patient and their caregivers) were randomised to a control group (RTG, *n* = 12) or an experimental group (CTG, *n* = 13). Participant dyads in the RTG group received routine teleoccupational guidance. Participant dyads in the CTG group received a six-step procedure: coaching-based teleoccupational guidance over 3 months via WeChat. Participant dyad compliance, the difficulty and suitability of outcome measures, and adverse effects were used to assess feasibility. The Reintegration to Normal Living Index, the Lawton Instructive Activities of Daily Life (Lawton IADL) scale, the Intrinsic Motivation Inventory, the Fugl–Meyer Assessment—Upper Extremity scale, the 6 min walking test, and the Stroke-Specific Quality of Life Scale were used to assess effectiveness outcomes of home-based stroke survivors; the Caregiver Benefit Finding Scale and the Zarit Caregiver Burden Interview were used to assess the effectiveness outcomes of family caregivers. Feasibility measures were assessed at the end of the pilot trial, and effectiveness measures were evaluated pre-intervention and post-intervention (after 3 months). The CTG programme significantly improved home-based stroke survivors’ participation in daily life, IADL score, and intrinsic motivation, and increased caregivers’ perceived benefit, and tended (not significantly) to reduce care burden. CTG has the potential to promote better integration of home-based stroke patients into their families and society, improve their quality of life and family well-being, and provide a reference for home rehabilitation of other clinical chronic diseases. CTG is a safe, effective, and promising intervention for home-based stroke populations and their caregivers and warrants further investigation in a larger randomised controlled trial.

## 1. Introduction

Stroke has become the leading cause of disability in adults worldwide [1,2]. Hemiplegia is one of the most common motor dysfunctions of stroke, which not only affects the quality of life (QoL) of patients but also brings a huge economic burden to families and society. Studies have shown that rehabilitation plays a crucial role for stroke survivors in promoting functional recovery, improving QoL, and gaining independence [3]. However, home-based stroke survivors often face difficulties in timely adjustment to rehabilitation plans, gaining adequate adherence to rehabilitation therapies, scientific stroke-related knowledge, and sufficient confidence and self-efficacy in the rehabilitation process [4,5,6], which limit patients’ return to normal family and social life. Therefore, positive and effective support services for stroke rehabilitation are needed to solve these problems and help home-based stroke survivors participate smoothly in family and societal life.

Coaching is a multi-dimensional, cognitive behavioural intervention that promotes patient-centred, goal-oriented development through various strategies, such as interviews, content education, and the inculcation of other skills to enhance self-monitoring behaviours and a sense of responsibility, cultivation of internal motivation, skill acquisition, and to enhance the well-being of individuals and facilitate the achievement of their health-related goals [7,8]. Motivational interviewing such as emotional support, expressing empathy, supporting self-efficacy, looking forward, looking back, and so on are core components of coaching strategies that motivate patients to achieve their goals of improved quality of life and better recovery outcomes. Currently, coaching has been employed mainly in managing chronic diseases, such as stroke and diabetes; coaching could effectively help patients with chronic diseases gain motivation and self-efficacy in self-management and gain the ability to deal with various problems in their diseases [9,10,11].

In recent years, coaching has gradually been applied in home-based stroke occupational therapy, and published research results show that the combination of coaching and occupational therapy can mobilise the internal motivation and innovative thinking of home-based stroke patients, and improve occupational performance, self-management, and compliance [12,13,14]. Many fundamental coaching principles are congruent with occupational therapy values and commitment to client-centred practice. However, most studies lacked structured and systematic coaching interventions and failed to address the important role of family caregivers in stroke rehabilitation. Therefore, we developed a coaching-based teleoccupational guidance (CTG) protocol that systematically integrated coaching into occupational therapy, constructed a six-step structured intervention process, and included family caregivers in the intervention to improve home-based stroke patients’ participation in daily life, instructive activities of daily living (IADL), intrinsic motivation, QoL, and caregivers’ perceived benefits, and to relieve care pressure on families.

The aim of this pilot study was to investigate the feasibility and effectiveness of a 3-month CTG programme for home-based stroke survivors and their family caregivers. The feasibility objectives were to examine the compliance of participant dyads, the difficulty and suitability of outcome measures, and potential adverse effects. The effectiveness objectives were to explore the effects of CTG in stroke survivors’ participation ability in daily life, IADL, intrinsic motivation, motor function, QoL, caregivers’ perceived benefit, and caregiver-related burden. We hypothesised that the CTG programme would be feasible to conduct and could improve the abovementioned indicators. Additionally, through this pilot study, the authors wanted to provide useful suggestions for future larger randomised controlled trials.

## 2. Materials and Methods

### 2.1. Study Design

An assessor-blind pilot randomised controlled study was conducted. Participants were recruited from the Department of Rehabilitation Medicine of Hebei General Hospital. This pilot study followed the Consolidated Standards of Reporting Trials (CONSORT) checklist [15] to guarantee methodological quality (see Supplemental Materials). The Ethics Committee of Hebei General Hospital approved the study (approval number: 2022 Scientific Research LunShen, No. 125), and informed consent was obtained from all participants before their enrolment in the study. The study followed the Declaration of Helsinki and Good Clinical Practice and was registered in the Chinese Clinical Trial Registry (ChiCTR2200061107).

### 2.2. Study Participants

Stroke survivors and their family caregivers as participant dyads (each participant dyad consisted of one home-based stroke patient and their primary caregivers) were recruited in this trial. Inclusion criteria for the stroke survivors were as follows: (a) men or women aged 18 to 70 years; (b) diagnosed with ischaemic or haemorrhagic stroke, either by computerised tomography scanning or magnetic resonance imaging; (c) more than 6 months after the onset of stroke; (d) modified Rankin scale (MRS) score of 2 to 4 points; (e) in home rehabilitation, and with no admission plan within 3 months; and (f) resided within or around the city of Shijiazhuang. Exclusion criteria for the stroke survivors were as follows: (a) Glasgow coma scale (GCS) score of less than 15; (b) impaired cognition (Mini-Mental State Examination < 21); (c) severe comorbidities, including circulatory, digestive, immune, and haematological disorders; (d) other diseases of the locomotor system, such as fracture, severe osteoporosis, and osteoarthritis, which could influence motor function; (e) bilateral brain lesions; and (f) severe sensory dysfunction.

Furthermore, the inclusion criteria for family caregivers were as follows: (a) men or women aged >18 years; (b) were the primary caregivers, who could ensure the time and support of participating in the patient’s home rehabilitation; and (c) had the ability to operate WeChat.

### 2.3. Randomisation and Blinding

After signing the informed consent form and completing the baseline assessment, computer-generated block random numbers (blocks of four) were used to randomly assign the participant dyads to either the intervention or control group. Allocation concealment was implemented using opaque envelopes that were numbered and sealed. A third-party, independent researcher, who was not involved in this pilot study, conducted the entire randomisation process.

### 2.4. Interventions

#### 2.4.1. Coaching-Based Teleoccupational Guidance (Intervention Group)

Over 3 months, participant dyads received a six-step coaching cycle intervention as follows: (a) building coaching relationships; (b) guiding participant dyads to identify up to five occupational goals; (c) developing and concretising occupational plans; (d) carrying out teleoccupational plans; (e) concluding experience; and (f) generalising experience and entering the next cycle. Occupational therapists provided participants with a weekly WeChat coaching video session (40–60 min each) during the intervention period, which was guided by the “ORAG” (Open-ended questions, Reflective listening, Affirmation, and Guide) programme. The weekly WeChat coaching session allowed timely evaluation of the completion of identified goals and adjustment of intervention plans. If patients completed identified goals, the occupational therapist used the Canadian Occupational Performance Measure (COPM) to explore goals with participant dyads and start a new CTG cycle. Caregivers play dual roles of “coachees” and “coaches”. As “coachees”, occupational therapists provide related coaching strategies to caregivers to train and improve the ability of coaching; as “coaches”, caregivers use this coaching knowledge to assist stroke patients in making changes towards identified goals. Details of the CTG protocol are described elsewhere [16].

#### 2.4.2. Routine Teleoccupational Guidance (RTG, Control Group)

Participant dyads received home-based occupation plans according to the selected occupational goals of the stroke survivors. Over 3 months, the dyads had a weekly teleoccupational video session (40–60 min each) with the occupational therapist via WeChat, which aimed to help the dyads adjust plans and solve problems. Caregivers only played the role of monitoring and care in the control group.

### 2.5. Outcome Measures

All assessments were conducted by an experienced occupational therapist who was not involved in the randomisation and intervention. Feasibility measures were assessed at the end of the pilot trial and effectiveness measures were evaluated pre-intervention and post-intervention (after 3 months).

#### 2.5.1. Feasibility Measures

The feasibility measures for the CTG program were as follows: (a) compliance of participant dyads, including rates of retention, assessment attendance, coaching session completion, and satisfaction with using WeChat; (b) the difficulty and suitability of outcome measures; and (c) adverse effects.

#### 2.5.2. Effectiveness Outcome Measures

Effectiveness outcome measures for home-based stroke survivors were as follows:

- Participation was assessed using the Reintegration to Normal Living Index (RNLI), which is a self-reported satisfaction scale to test participation from the perspective of physical activities and social events. The RNLI has been translated into Chinese and has been proven reliable and valid for the Chinese population with Cronbach’s α of 0.92 [17];
- IADL were assessed using the Lawton Instructive Activities of Daily Life (Lawton IADL) scale [18], which tests instructive activities of daily living (ADLs) from the perspective of using the telephone, shopping, preparing food, housekeeping, doing laundry, using transportation, handling medications, and handling finances, and which has been widely applied to Chinese stroke survivors;
- Intrinsic motivation was assessed using the Intrinsic Motivation Inventory (IMI) [19], which is a self-reported scale to test motivational structures for targeted activities from perspectives of interest/enjoyment, perceived competence, pressure/tension, and value/usefulness;
- Motor function was assessed using the Fugl–Meyer Assessment—Upper Extremity (FMA-UE) scale and the 6 min walking test (6MWT) [20,21]. The FMA-UE scale is used to assess the movement, coordination, and reflex actions of the shoulder, elbow, forearm, wrist, and hand; it is an important indicator for evaluating motor function of the upper extremity with good reliability and validity. The 6MWT refers to the walking distance of patients in 6 min, which can reflect the walking capacity of stroke survivors in home environments. The FMA-UE scale and the 6MWT have been widely applied to Chinese stroke survivors;
- QoL was assessed using the Stroke-Specific Quality of Life Scale (SS-QOL), which is a 49-item self-reported test of QoL regarding energy, family roles, language, mobility, mood, personality, self-care, social roles, thinking, upper extremity function, vision, and work/productivity. The SS-QOL has been translated into Chinese and has good internal consistency with Cronbach’s α for each domain ranging from 0.63 to 0.90 [22].

Effectiveness outcome measures for caregivers were as follows:

- Perceived benefits were assessed using the Caregiver Benefit Finding Scale (CBFS). The CBFS was designed by Chinese scholars, with a Cronbach’s α range of 0.885 to 0.953 for the subscales, and is a self-reported scale to test caregivers’ perceived benefits from perspectives of individual growth, health promotion, family growth, and self-sublimation [23];
- Caregiver-related burden was assessed using the Chinese version of the Zarit Caregiver Burden Interview (ZBI-c), which is a 22-item self-reported scale evaluating caregiver burden from the dimensions of personal strain and role strain. The ZBI-c was validated in the Chinese population with a Cronbach’s α of 0.87 [24].

### 2.6. Statistical Analyses

SPSS statistical software version 27.0 (SPSS Inc., Chicago, IL, USA) was used to perform statistical analyses. Descriptive analyses (mean ± standard deviation [SD] or frequency) were used to analyse the demographic data in each group. All data were tested for normality of distribution using the Shapiro–Wilk test. The paired-sample t test or the Wilcoxon signed-rank test was used to analyse within-group differences. The independent *t*-test, Mann–Whitney U test, or χ^2^ test was used to analyse possible differences between groups from baseline to post-intervention and reported mean ± SD, 95% confidence intervals (CIs), and *p* values. The level of statistical significance was set at 0.05. The effect size was described with Cohen’s d statistic, which was calculated by the mean and SD of the parameters. Cohen’s d < 0.2, 0.2 < d < 0.5, 0.5 < d < 0.8, and d > 0.8 were respectively considered “trivial”, “meaningful but small”, “moderate”, and “large” effect sizes.

### 2.7. Sample Size

According to Whitehead’s suggested sample size for a pilot study [25], we planned to recruit 30 participant dyads (30 stroke survivors and 30 caregivers).

## 3. Results

### 3.1. Demographic Data

**Figure 1:**
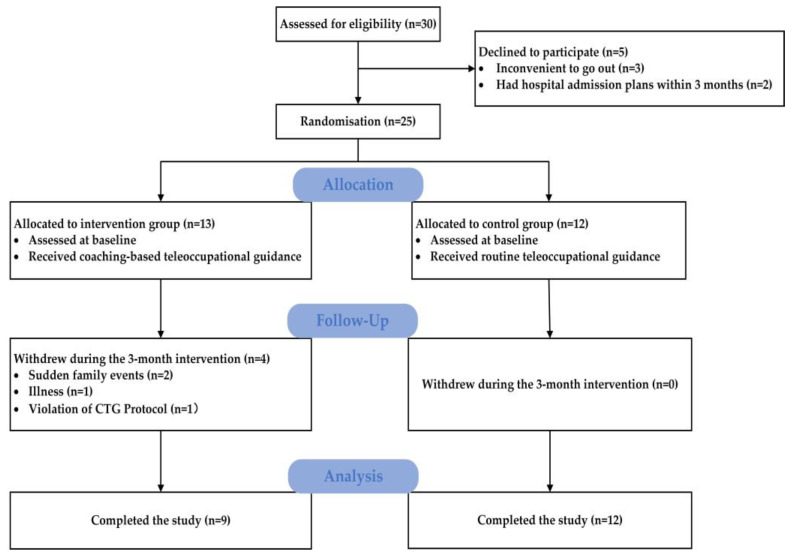
Flow chart of the study design.

Of the 30 participant dyads identified as eligible for the pilot study between January 2022 and 25 May 2022 (83.3%) agreed to participate. Included research participants were randomised into either the CTG group (*n* = 13) or the RTG group (*n* = 12). Twenty-one participant dyads completed the pilot study (Figure 1). At baseline, all demographic, clinical characteristics and effectiveness outcome measures for both groups had no statistically significant differences, as shown in Table 1 (*p* > 0.05). 

### 3.2. Feasibility Objectives

Twenty-one participant dyads (84%) completed the 3-month pilot study, and four dyads in the CTG group withdrew from the study: two dyads withdrew because of unforeseen family events (other family members were sick, which meant caregivers had insufficient time to look after patients); one dyad withdrew because the stroke survivor was admitted to hospital for acute appendicitis; and one patient was stubborn, did not want to make changes, and quit the intervention. The remaining 21 participant dyads (84%) completed 12 coaching sessions. Assessment attendance (84%) was the same as the rate of coaching sessions. There were no reported technical difficulties or privacy breaches when using WeChat.

Participant dyads completed all outcome measures with ease. However, 15 of 21 stroke patients (71.4%) had difficulty completing the IMI scale, which adopts positive and negative questioning methods for the same question: for example, “I felt very tense while doing this activity” and “I was very relaxed in doing these”; such questioning made stroke patients confused and made it hard for them to rationally score the IMI scale. Researchers found the problem and gave detailed explanations for these patients to correctly score and successfully complete the IMI assessment. All outcome indicators were matched with the aims of the pilot study, but there was a lack of quantitative data measuring how participants made changes during the intervention. No adverse events were observed during the study period.

### 3.3. Effectiveness Assessments

Table 2 shows the pre-test and post-test study outcomes for the CTG and RTG groups (within-group comparison). The CTG group showed significant changes in terms of the pre-test and post-test scores in all effectiveness assessments; effect sizes were large for RNLI (Cohen’s d 1.066), Lawton IADL (2.227), and ZBI-c (2.071), medium for IMI (0.530), SS-QOL (0.517), and CBFS (0.780), small but meaningful for the 6MWT (0.388), and trivial for the FMA-UE (0.125). The RTG group showed significant changes in terms of the pre-test and post-test scores in all effectiveness assessments; effect sizes were large for Lawton IADL (Cohen’s d 0.809), CBFS (1.024), and ZBI-c (1.369), medium for RNLI (0.694) and IMI (0.648), small but meaningful for the 6MWT (0.353) and SS-QOL (0.466), and trivial for the FMA-UE (0.082).

Table 3 shows the mean changes from pre-test to post-test for study outcomes between the CTG and RTG groups (between-group comparison). Compared to the RTG group, the CTG group showed statistically significant mean changes in RNLI, Lawton IADL, IMI, and CBFS; effect sizes were large for Lawton IADL, IMI, and CBFS, and small but meaningful for the RNLI. The inter-group analysis of FMA-UE, 6WMT, SS-QOL, and ZBI-c revealed no statistically significant differences.

## 4. Discussion

To the best of our knowledge, this is the first study investigating the feasibility and effectiveness of coaching combined with teleoccupational guidance in home-based stroke survivors and their caregivers. The findings of the pilot study support that CTG is feasible to conduct, is acceptable, and shows statistically significant changes in home-based stroke patients’ participation ability in daily life, instructive ADLs, intrinsic motivation, and caregivers’ perceived benefits.

Regarding feasibility of the pilot study, overall recruitment (83.3%), retention rates (84.0%), and coaching sessions and assessment attendance (84.0%) were acceptable, and the use of WeChat was equally satisfactory. The CTG group lost three participant dyads because of unexpected family events and other illnesses, which were unrelated to the intervention, and one stroke survivor withdrew from the study due to a stubborn personality; this patient refused to accept professional advice and insisted on following their own ideas that lacked science and security for rehabilitation, which indicates that patients’ personalities may influence the efficacy of coaching. Therefore, the authors suggest that personality assessment questionnaires, such as the Eysenck Personality Questionnaire, should be included in the initial evaluation of stroke patients to obtain an idea of patients’ personality characteristics. Furthermore, outcome measures were easy to complete and matched the aims of the pilot study, although clearer instructions for using the IMI were needed. The authors also suggest that coaching diaries should be added to the effectiveness measures to observe the efforts of stroke patients during the intervention. No adverse events were reported in this study.

For home-based stroke survivors’ participation ability, RNLI scores significantly improved in both groups after the intervention compared to pre-intervention (*p* < 0.001), but the CTG group showed significantly greater improvement than the RTG group (*p* = 0.013). The World Health Organization defines participation as “the ability of a person to engage in life situations and meaningful activities” [26]. Most home-based stroke survivors have varying degrees of difficulty in participating in instrumental ADLs, leisure, and community activities; such difficulties reduce patients’ independence, increase social isolation, anxiety, and depression, and increase the burden of care for families [27]. Both the CTG and RTG groups used COPM to guide the development of home-based occupational goals that revolved around ADLs, productivity, and leisure activities, while these goals were closely related to the RNLI evaluation indicators. Meanwhile, the occupational therapist used the COPM tool to dynamically assess the progress of stroke patients’ occupational goals in a timely manner during weekly WeChat coaching sessions/teleoccupational meetings. Thus, the attainment of occupational goals in stroke patients was visually reflected in the improvement of RNLI scores in both groups. In addition, the reason for the significant difference in RNLI in the CTG group versus the RTG group may be that CTG could provide coaching strategies and coaching sessions for home-based stroke survivors to help patients actively find problems, identify barriers, analyse reasons, and explore feasible solutions to gain self-efficacy and independent problem-solving ability when facing future participation problems.

Notably, the effect size for RNLI was 0.360, which indicates that CTG statistically significantly improves the participation of home-based stroke patients, although this is not as clinically significant. One factor that may have influenced the effect size is that although the RNLI has become an important outcome measure that evaluates stroke patients’ return to families and society [28], participation is a complex concept and structure [29]. The RNLI only has 10 items to assess stroke patients’ participation and uses self-reported satisfaction assessments, which makes it subjective in its evaluation, relatively thin in content, and hard to cover all elements of participation and objectively perceive changes. Therefore, a future, larger randomised controlled trial should consider a composite and objective outcome measure to evaluate participation changes.

For instructive ADLs, Lawton IADL scores improved in both groups after the intervention compared to pre-intervention (*p* < 0.001), but the CTG group showed significantly greater improvement than the RTG group (*p* < 0.001) and had a large effect size (Cohen’s d: 1.416). As mentioned earlier, both groups focused on ADLs and used occupational strategies to help home-based stroke survivors improve their independent survival ability, which increased Lawton IADL scores in the CTG and RTG groups. Possible factors contributing to the significantly greater improvement in IADL scores and the large effect size in the CTG group might be that coaching motivated the home-based stroke population to actively think and acquire problem-solving methods influencing IADLs by setting goals, providing related knowledge and empowerment, and adjusting occupational activities; such changes may have helped patients to develop and improve proactivity and confidence to courageously face and solve future problems influencing IADLs.

For intrinsic motivation, IMI scores improved in both groups after the intervention compared to pre-intervention (*p* < 0.001), but the CTG group showed a significantly greater improvement than the RTG group (*p* = 0.010) and had a large effect size (Cohen’s d: 1.223). An explanation could be that the act of reflecting on and selecting individual occupational goals through COPM may help stroke survivors realise the gap between themselves and their goals, and may help patients take action to achieve their goals; such action may contribute to progress in the CTG and RTG groups. This explanation is in line with results from a previous study [30], which showed that goal setting, by itself, may promote goal attainment for stroke populations. Furthermore, important factors influencing the significant improvement and large effect size for IMI in the CTG group might be that strategies of coaching, such as motivational interviews, reflective listening, looking forwards, and looking back, help occupational therapists perceive emotional changes in participants (and explore the potential reasons) to build participants’ internal motivation and self-efficacy for facing difficulties, while weekly structured coaching sessions can ensure the quality of coaching and encourage home-based stroke survivors to keep gaining momentum. Additionally, caregivers play a crucial role in improving the intrinsic motivation of home-based stroke survivors because caregivers spend a lot of time with patients and can help patients maintain motivation promptly through the coaching strategies taught by the occupational therapist.

For motor function, although the CTG and RTG groups showed significant changes in terms of the pre-test and post-test scores in FMA-UE, effect sizes were trivial. No significant improvement in the FMA-UE score was obtained in the between-group comparison. Several factors may have contributed to this negative result. First, researchers recruited home-based stroke survivors more than 6 months after stroke onset. Previous studies have shown that stroke survivors typically experience a motor recovery plateau after 6 months, indicating slow (or no further) motor function improvements after this time [31,32,33]. Second, the intervention protocol put the highest priority on the occupational performance of identified goals and the ability to participate in normal life, which meant a relatively reduced focus on motor function exercises per se. Moreover, 6MWT distance improved in both groups after the intervention compared to pre-intervention (*p* < 0.001), but there was no statistically significant difference between the two groups (*p* = 0.08). This pilot study shows that home-based stroke survivors in both groups pay particular attention to walking capacity and are keen to get out because they believe that the ability to get out of home is a sign of recovery, which can bring them pride and a sense of achievement.

For QoL, SS-QOL scores improved in both groups after the intervention compared to pre-intervention (*p* < 0.001), but there was no statistically significant difference between the two groups (*p* = 0.443). Teleoccupational guidance has the advantage of designing individual occupational therapy programmes according to the needs of stroke survivors and their home environments, integrating function into patients’ lives, which can greatly improve ADLs and QoL. However, the SS-QOL scale is a 49-item self-reported scale that also addresses dimensions of language, emotion, personality, thinking, and visual acuity, which indicates that the scale may lack sufficient sensitivity for this pilot study. Therefore, the authors recommend that future, larger randomised controlled trials should choose a QoL scale that focuses more on ADLs in home-based stroke survivors.

For caregivers’ perceived benefit, CBFS scores improved in both groups after the intervention compared to pre-intervention (*p* < 0.001), but the CTG group showed significantly greater improvement than the RTG group (*p* = 0.010) and had a large effect size (Cohen’s d: 1.236). Perceived benefit is defined as a cognitive and behavioural coping process in which individuals find personal, social, psychological, and spiritual benefits from negative life events [34]. Stroke caregivers bear heavy mental pressure, such as anxiety and depression, in the care of stroke survivors, which reduces caregiver QoL and can affect physical health [35]. In this pilot study, researchers used coaching strategies, such as individual education, motivational interviews, and metacognition, to guide caregivers to face difficulties with a positive perspective, and to highlight positive psychological feelings and encourage caregivers to acknowledge and praise the gains from this caring process [36], which may account for the significant improvement and large effect size for caregivers’ perceived benefit in the CTG group. Furthermore, caregivers with high perceived benefits could motivate stroke survivors to adopt an optimistic mindset and take positive action to achieve goals.

For caregiver-related burden, ZBI-c score was reduced in both groups after the intervention compared to pre-intervention (*p* < 0.001), but there was no statistically significant difference between the two groups (*p* = 0.067). Several reasons may contribute to the improvement in both groups. First, teleoccupational guidance provided care knowledge and skills for caregivers: for example, modification of the home environment according to the needs of the participant dyads, such as the installation of bidet toilets and grab bars in toilets, the addition of non-slip mats on toilet and kitchen floors, a reduction in the number of steps and thresholds indoors, and the adjustment of kitchen preparation table heights; these changes could not only ensure living convenience and safety for home-based stroke survivors but could also help caregivers to more easily look after patients and decrease the care burden. Second, caregivers were invited to set goals and explore occupational plans with stroke survivors in both groups, and these goals and plans were closely related to reducing the care burden for caregivers. So, when stroke survivors’ ADLs and participation improved, the caregiver-related burden naturally decreased. However, the authors also endorse the potential efficacy of the CTG in reducing caregiver-related burden. Caregivers need to continuously learn caregiving skills and coaching strategies during the intervention cycle, which can be burdensome and stressful for caregivers; but when caregivers convert what they learn into their own competencies, the burden of learning and caregiving will be greatly reduced. No follow-up was performed in this pilot study, so the potential differences in caregiver-related burden may not have been observed. Additionally, given the small sample size, the between-group difference for ZBI-c was of borderline statistical significance (*p* = 0.067), combined with a large effect size (Cohen’s d: 1.370), and may still indicate potential benefits for CTG in reducing care burden that warrant further study by expanding the sample size and adding a 3-month follow-up.

This pilot study has several limitations. First, because of the small sample size, the results should be interpreted cautiously when generalising them to home-based stroke populations. Second, it is difficult to conduct a double-blind study due to the nature of the intervention, which may lead to Hawthorne effects: the tendency to perform or perceive differently when one knows they are being observed. Third, there may have been instances where some coaching strategies, such as emotional support, were provided to participants in the control group; this was inevitable for the treating therapist because of professional ethics, but may have increased the risk of bias.

## 5. Recommendations for Future Studies

According to the results of this study, some alterations should be made to ensure a successful, larger randomised controlled trial: (a) add personality assessment questionnaires, such as the Eysenck Personality Questionnaire, into the inclusion criteria; (b) provide clearer and more detailed instructions for using the IMI; (c) add a quantitative indicator: e.g., coaching diaries; (d) choose a composite and objective outcome measure to evaluate participation changes; (e) choose a QoL scale that focuses more on ADLs; and (f) add a 3-month follow-up.

## 6. Conclusions

This pilot randomized controlled trial demonstrated that CTG could improve home-based stroke survivors’ participation, IADLs, and intrinsic motivation, and could increase caregivers’ perceived benefit and reduce care burden. Overall, the trial suggests that CTG is a safe, effective, and promising intervention for home-based stroke populations and caregivers that now warrants further investigation in a larger randomised controlled trial. Future studies are needed to explore the efficient coaching model for home-based stroke patients and determine the characteristics of the most suitable stroke population for the coaching.

## Figures and Tables

**Table 1:** Basic characteristics and effectiveness outcome measures for stroke survivors and caregivers.

Variable	CTG (*n* = 13)	RTG (*n* = 12)	*p* Value
Age (years)	51.31 ± 8.56	53.25 ± 5.75	0.514 ^a^
Sex (male/female)	12/1	10/2	0.490 ^b^
Type of stroke (ischaemic/haemorrhagic)	7/6	8/4	0.513 ^b^
Paretic side (left/right)	10/3	7/5	0.319 ^b^
Months after stroke	10.31 ± 3.82	10.92 ± 2.07	0.629 ^a^
Education			
Lower than high school	4	6	0.602 ^c^
High school	5	2	
Higher education or higher	4	4	
Modified Rankin Scale			
2, Slight disability	0	1	0.298 ^c^
3, Moderate disability	13	11	
4, Moderately severe disability	0	0	
Caregiver Role			
Couple	12	11	0.367 ^b^
Child	1	0	
Siblings	0	1	
Caregiver education level			
Lower than high school	6	5	0.403 ^c^
High school	3	0	
Higher education or higher	4	7	
Outcome measures			
Stroke survivors			
RNLI	54.00 ± 20.24	64.92 ± 23.38	0.277 ^a^
Lawton IADL	11.67 ± 4.21	13.92 ± 4.56	0.262 ^a^
IMI	143.67 ± 23.87	145.00 ± 10.72	0.865 ^a^
FMA-UE	36.67 ± 19.07	35.50 ± 12.44	0.867 ^a^
6MWT	129.00 ± 59.36	126.08 ± 56.27	0.910 ^a^
SS-QOL	166.33 ± 27.72	160.42 ± 29.94	0.649 ^a^
Caregivers			
CBFS	92.33 ± 22.80	95.00 ± 10.87	0.725 ^a^
ZBI-c	43.67 ± 9.37	40.33 ± 11.00	0.474 ^a^

^a^ Independent samples *t*-test; ^b^ Chi-squared test; ^c^ Mann–Whitney U test. Values are presented as mean ± standard deviation or frequency; CTG: coaching-based teleoccupational guidance; RTG: routine teleoccupational guidance; RNLI: Reintegration to Normal Living Index; Lawton IADL: Lawton Instructive Activities of Daily Life; IMI: Intrinsic Motivation Inventory; FMA-UE: Fugl–Meyer Assessment—Upper Extremity; 6MWT: 6 min walking test; SS-QOL: Stroke-Specific Quality of Life; CBFS: Caregiver Benefit Finding Scale; ZBI-c: Chinese version of the Zarit Caregiver Burden Interview.

**Table 2:** Pre-test and post-test effectiveness outcome measures in the intervention and control groups.

Variable	CTG (*n* = 9)				RTG (*n* = 12)			
	Pre-Test	Post-Test	*p* Value	Cohen’s d	Pre-Test	Post-Test	*p* Value	Cohen’s d
	Mean ± SD				Mean ± SD			
RNLI	54.00 ± 20.24	77.56 ± 23.68	<0.001	1.066	64.92 ± 23.38	79.33 ± 17.79	<0.001	0.694
Lawton IADL	11.67 ± 4.21	20.33 ± 3.54	<0.001	2.227	13.92 ± 4.56	17.08 ± 3.12	<0.001	0.809
IMI	143.67 ± 23.87	156.67 ± 25.16	<0.001	0.530	145.00 ± 10.72	152.17 ± 11.40	<0.001	0.648
FMA-UE	36.67 ± 19.07	36.67 ± 19.04	0.028	0.125	35.50 ± 12.44	36.50 ± 11.90	0.002	0.082
6MWT	129.00 ± 59.36	152.33 ± 60.95	<0.001	0.388	126.08 ± 56.27	145.67 ± 54.86	<0.001	0.353
SS-QOL	166.33 ± 27.72	180.89 ± 28.58	<0.001	0.517	160.42 ± 29.94	173.92 ± 28.03	<0.001	0.466
CBFS	92.33 ± 22.80	108.89 ± 19.56	<0.001	0.780	95.00 ± 10.87	106.42 ± 11.72	<0.001	1.024
ZBI-c	43.67 ± 9.37	24.56 ± 9.08	<0.001	2.071	40.33 ± 11.00	25.42 ± 10.78	<0.001	1.369

CTG: coaching-based teleoccupational guidance; RTG: routine teleoccupational guidance; RNLI: Reintegration to Normal Living Index; Lawton IADL: Lawton Instructive Activities of Daily Life; IMI: Intrinsic Motivation Inventory; FMA-UE: Fugl–Meyer Assessment—Upper Extremity; 6MWT: 6 min walking test; SD: standard deviation; SS-QOL: Stroke-Specific Quality of Life; CBFS: Caregiver Benefit Finding Scale; ZBI-c: Chinese version of the Zarit Caregiver Burden Interview.

**Table 3:** Mean changes from pre-test to post-test for effectiveness outcome measures between the intervention and control groups.

Variable	CTG (*n* = 9)	RTG (*n* = 12)	*p* Value	ED, CTG vs. RTG (95% CI)	Cohen’s d
	Pre-Test to Post-Test	Pre-Test to Post-Test			
	Mean ± SD	Mean ± SD			
RNLI	23.56 ± 6.98	14.42 ± 7.95	0.013 *	9.14 (2.16–16.11)	0.360
Lawton IADL	8.67 ± 3.94	3.17 ± 2.33	<0.001 *	5.50 (2.63–8.87)	1.416
IMI	13.00 ± 5.55	7.17 ± 3.83	0.010 *	5.83(1.56–10.11)	1.223
FMA-UE	1.00 ± 1.12	1.00 ± 0.85	1.00	0.00 (−0.90 to 0.90)	0.000
6MWT	23.33 ± 5.98	19.58 ± 3.34	0.08	3.75 (−0.53 to 8.03)	0.774
SS-QOL	14.56 ± 1.59	13.50 ± 3.78	0.443	1.06 (−1.76 to 3.87)	0.302
CBFS	16.56 ± 4.69	11.42 ± 3.55	0.010 *	5.14 (1.38–8.90)	1.236
ZBI-c	−19.11 ± 4.76	−14.92 ± 4.98	0.067	−4.19 (−8.71 to 0.32)	1.370

CTG: coaching-based teleoccupational guidance; RTG: routine teleoccupational guidance; ED: estimated difference; RNLI: Reintegration to Normal Living Index; Lawton IADL: Lawton Instructive Activities of Daily Life; IMI: Intrinsic Motivation Inventory; FMA-UE: Fugl–Meyer Assessment—Upper Extremity; 6MWT: 6 min walking test; SS-QOL: Stroke-Specific Quality of Life; CBFS: Caregiver Benefit Finding Scale; ZBI-c: Chinese version of the Zarit Caregiver Burden Interview; SD: standard deviation. * *p* < 0.05 between groups.

## Data Availability

The data that support the findings of this study are available from the corresponding author upon reasonable request.

## Supplementary Materials

The following supporting information can be downloaded at: https://www.mdpi.com/article/10.3390/ijerph192316355/s1, CONSORT 2010 checklist.
